# mRMC: A rotating multi-gene pyramiding scheme based on the principle of minimum rotation and maximum complementarity in animals

**DOI:** 10.1186/s12711-026-01112-2

**Published:** 2026-11-11

**Authors:** Ruihan Mao, Yue Zhuo, Heng Du, Wanying Li, Lei Zhou, Li Jiang, Jian-Feng Liu

**Affiliations:** State Key Laboratory of Animal Biotech Breeding, Frontiers Science Center for Molecular Design Breeding (MOE), College of Animal Science and Technology, China Agricultural University, Beijing, 100193 China

## Abstract

**Background:**

Gene pyramiding is a breeding process used to combine multiple favorable alleles of target genes from different breeds into a single population. Existing strategies in plants aim to obtain a small number of “ideal individuals” and form inbred lines through asexual reproduction or inbreeding methods. However, animals exhibit distinct features, including sexual reproduction with low reproductive ability and intolerance to inbreeding. These limitations hinder the implementation of existing strategies in animals.

**Results:**

“Breeding by Design” is a concept that aims to strategically assemble known favorable alleles at major-effect loci of agronomic importance into breeding populations. To accelerate this process of in animals, we proposed a rotating scheme named “mRMC” (Minimum Rotation and Maximum Complementarity) as an effective design for multi-gene pyramiding. The mRMC is designed to match paternal lines with “the least rotation of labels (min-rotation) and the most complementary (max-complementarity)” genotype to form mating pairs. We compared this method with existing schemes in a simulation of breeding animals. The results showed that, to obtain all-target-gene carriers, mRMC reduced the required number of generations by three and one compared with cascading and noncascading pyramiding method, respectively. The average minor allele frequency (MAF) of all gene makers in the population under mRMC was the highest among all the schemes in each generation. The inbreeding level of the mRMC was lower and changed less throughout the entire process than the other pyramiding schemes. Additionally, upon completion of pyramiding, compared with the other two schemes, mRMC reduced the loss of genetic gain by 17.70% and 11.76%, respectively.

**Conclusions:**

Overall, the proposed mRMC demonstrated clear superiority over existing methods for animal breeding. This strategy will improve breeding schemes and facilitate studies on gene pyramiding in animals.

**Supplementary Information:**

The online version contains supplementary material available at https://doi.org/10.1186/s12711-026-01112-2.

## Background

With the rapid advancements in genetic technology, gene regions responsible for important economical traits can be identified and verified. Animals carrying favorable loci can be designed for performance improvement or disease resistance [1]. Over the past decades, marker assisted selection (MAS) has been employed to introduce favorable gene alleles or quantitative trait loci (QTL) into livestock breeding [2–5]. To balance short-term and long-term genetic response in breeding programs, many studies not only focus on the fixation of favorable alleles, but also raised the concern of selection index, genetic variation, genetic diversity, and average inbreeding level [6–9]. Focusing on specific-genotype and complex-trait GEBV selection, Kemper et al. [10] compared genotype-building with overall GEBV selection under a coancestry constraint in dairy cattle and found the latter more practical for substantial long-term progress in highly polygenic traits with low reproductive rates and low coancestry tolerance. The advent of gene editing technologies has opened new possibilities for animal breeding by enabling precise modification of specific genes beyond naturally occurring mutations [11–17]. “Breeding by Design” refers to a breeding strategy aimed at strategically assembling known favorable alleles into breeding populations. In animal breeding, where complex traits are typically under polygenic control, the scope of this concept is pragmatically limited to major-effect loci that have been identified and validated (e.g., through QTL mapping, GWAS, or gene editing studies). With advances in gene editing, pyramiding of such loci is becoming increasingly feasible for animal improvement. However, due to technological limitations, single-gene editing is relatively more practical than multi-gene editing [18], and large-scale gene editing for livestock production remains economically unfeasible. Consequently, a practical alternative is to edit a small number of animals with different genes as parents and spread the favorable alleles of target genes throughout the population via gene pyramiding [19].

Gene pyramiding was theoretically simulated [20–22] and practically applied in many plant breeding schemes [23–28], typically aiming to obtain at least one “ideal individual” carrying multiple favorable genes. Servin et al. conducted a relatively systematic and comprehensive theoretical framework for gene pyramiding in plants [29], which comprises two sequential steps: gene pyramiding and fixation. Here, “pyramiding” denotes the emergence of at least one individual that carries all target genes, whereas “fixation” refers to the stage at which every individual carries all target genes or the target genes have reached a certain frequency in the population. Thanks to their high reproductive capacity, short generation intervals, and tolerance to inbreeding, plants can readily develop inbred lines from limited individuals. Subsequently, Zhao et al. [30] and Xu et al. [31] extended this two-step framework to simulate animal populations, taking into account the distinct biological constraints of animals: long generation intervals and limited reproductive capacity. However, they offered no methodological innovations tailored specifically to outbred livestock populations.

Animals exhibit limited reproductive capacity, long generation intervals, obligate sexual reproduction, and intolerance to inbreeding. To achieve sustainable long-term genetic improvement in outbred animal populations, it is essential to maintain genetic diversity, preserve multiple paternal lines, and control inbreeding. Therefore, it is imperative to establish an efficient gene pyramiding strategy in order to produce trait-enhanced outbred animal populations.

In this study, we propose a novel rotating gene pyramiding strategy named “mRMC”, which is based on the principle of “Minimum Rotation and Maximum Complementarity”. In the proposed method, “Rotation” means that individuals from each paternal line are mated in turn with individuals from other lines. For example, individuals of line 1 might be crossed with line 2 in generation 1, with line 3 in generation 2, and with line 5 in generation 3, and so on in a rotating manner. “Complementarity” indicates that the two parents forming a mating pair carry different target-genotype subsets, each being a proper subset of the complete set of target genes, thereby maximizing the number of favorable alleles of target genes in the offspring. Therefore, “Maximum Complementarity” means that the mating pair is chosen to maximise the number of distinct target genes in their combined genotype (i.e., the size of the union of their target-gene sets). “Minimum Rotation” implies that, while maintaining this maximal complementarity, mating pairs are formed in a rotating manner across generations, with the smallest possible shift in labels from one generation to the next. Our simulation studies demonstrate that mRMC effectively improves pyramiding efficiency while maintaining genetic diversity and controlling inbreeding in outbred animal populations.

## Methods

To expand the available pyramiding strategy specialized for outbred animals, we proposed a new scheme using the mRMC method.

### mRMC: a newly proposed rotating pyramiding scheme

Suppose that we have $\:\mathrm{n}$ target genes and an initial population containing $\:\mathrm{n}$ paternal lines, where each line is homozygous for the favorable allele of one distinct target gene in the initial generation. In this study, a “paternal line” is defined as all individuals that can be traced back to the same founding sire; progeny sharing this ancestor are therefore said to originate from the same paternal line. We labelled the target gene number as the line number of the individuals in generation 0. The final goal of this pyramiding step is to accumulate all the genes in the whole population. To align with practical conditions, the objective is that every individual carries all favorable target alleles, without requiring complete homozygosity.

In our mRMC strategy, to form the best mating pairs, in generation * t*, the superior maternal parent $\:{\mathrm{p}}_{\mathrm{i}}^{\mathrm{*}}$ of paternal parent $\:\mathrm{i}$ is

1 $$\:{p}_{i}^{*}=\left\{\begin{array}{c}{p}_{i},\:{\:p}_{i}\le\:n\\\:\:\\\:{p}_{i}\:mod\:n,{\:p}_{i}>n\end{array}\right.$$

where

2 $$\:{p}_{i}\:=\:\left\{\begin{array}{c}i+{2}^{t},\:{2}^{t+1}\le\:n\\\:i+1,\:{2}^{t+1}>n\end{array}\right.\:$$

**Fig. 1:**
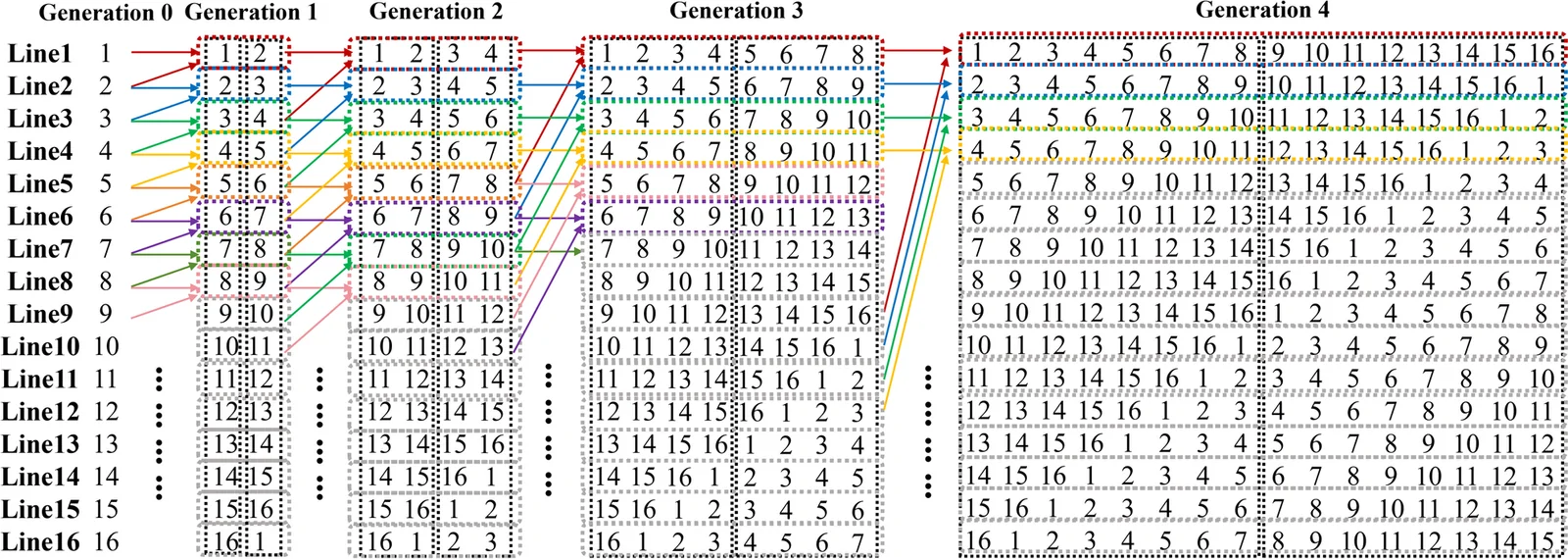
Rotating pyramiding scheme based on a rotational mating design (using sixteen target genes as an example due to layout limitations). Initially, suppose each paternal line contains one target gene, and the label of paternal line number corresponds to the target gene number. The notation [$\:\mathrm{t}$, $\:\mathrm{i}$] represents the $\:\mathrm{i}$-th paternal line in the $\:\mathrm{t}$-th generation. The arrows indicate the mating strategy, with the numbers in each row representing the target genes carried by the $\:\mathrm{i}$-th paternal line. After four generations of pyramiding, each paternal line accumulates a total of sixteen target genes

We assumed that the$\:\text{}\mathrm{i}$-th paternal line in the $\:\mathrm{t}$-th generation is represented by $\:\left[\mathrm{t},\mathrm{i}\right]$ (Fig. 1). Two different target genes can be combined simultaneously through a single mating. Therefore, it is obvious that, in the $\:\mathrm{t}$-th generation, for the $\:\mathrm{i}$-th paternal line, the pyramiding of genes labeled from $\:\mathrm{i}$ to $\:\mathrm{i}\mathrm{+}{\mathrm{2}}^{\mathrm{t}}\mathrm{-1}$ was completed. The next step was to identify the effective breeding pairs for producing the next generation. Therefore, the paternal line number with minimum rotation and maximum complementarity to the $\:\mathrm{i}$-th paternal line is located at $\:\mathrm{i}\mathrm{+}{\mathrm{2}}^{\mathrm{t}}$. If the situation of $\:{\mathrm{2}}^{\mathrm{t+1}}\mathrm{>n}$ occurs in generation $\:\mathrm{t}$, then any two genotypes are completely complementary in forming a mating pair to produce the ideal offspring carrying all favorable alleles. To simplify the mating plan, two lines whose label are numerically adjacent were set for mating. Besides, to obtain ideal individuals with all target genes, the total number of pyramiding generations ($\:\mathrm{T}$) needs to be the minimum integer satisfying ${\rm{T}} \ge \log_2\left( n \right)$.

**Fig. 2:**
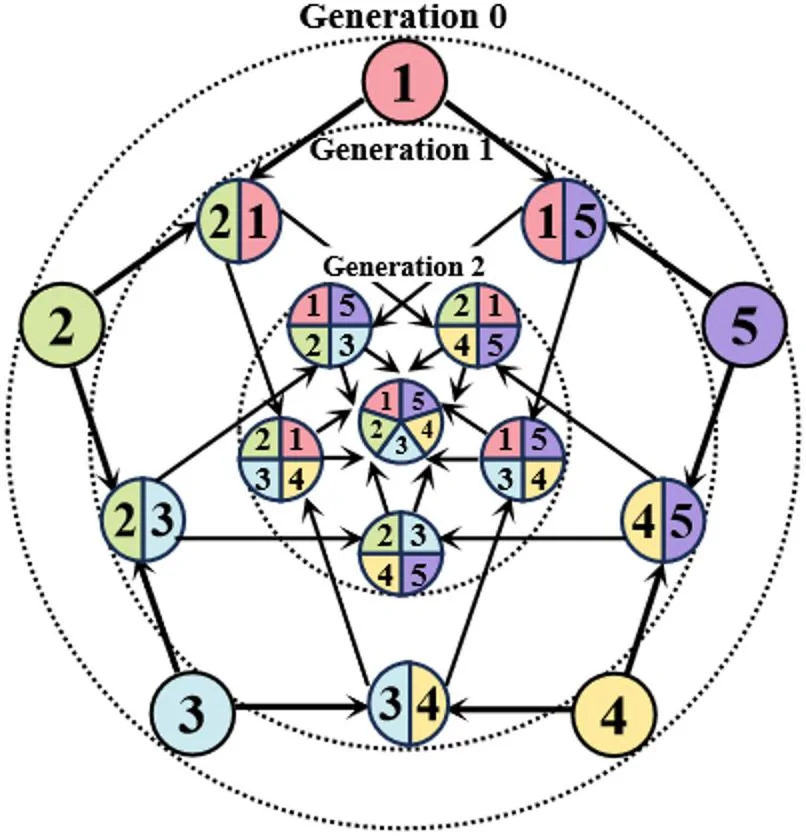
Rotating pyramiding scheme for cumulating five target genes. Each circle represents one paternal line, and the arrow indicates mating strategy. The numbers within the circles represent the labels of the target genes. The ultimate goal is to generate a population containing all five target genes through several generations of gene pyramiding. In generation *t*, paternal line *i* is mated with line *i* + 2^*t*^ as long as 2^*t* + 1^ ≤ *n*, and with the adjacent line when 2^*t* + 1^ > *n* (*n* = 5, the number of target genes), so the mating step doubles in successive generations (1, 2, 4, …) and restarts from 1 once it exceeds *n*. Consequently, mating pairs are formed between adjacent lines in generation 0, between lines one position apart in generation 1, and between adjacent lines again in generation 2 (2^3^ = 8 > *n*), which is why the scheme is termed “rotating”. After three generations of pyramiding every individual carries all five target genes, and the ultimate goal is a population containing all favourable alleles of the five target genes

Taking the five target genes as an example, the breeding objective was to develop a population possessing all five genes simultaneously. Graphically, these five paternal lines were arranged in the shape of a regular pentagon (Fig. 2). In the initial generation (generation 0) of the pyramiding process, mating pairs were formed from two adjacent paternal lines. In the subsequent generation (generation 1), the mating pairs consisted of two paternal lines that were one position apart. In generation 2, the mating pairs returned to adjacent paternal lines. Hence, this strategy is termed “rotating” pyramiding scheme.

This scheme is derived from the rotational mating design used in large multibreed hybrid populations, where the even distribution of target genes among individuals helps control the population inbreeding level and maintain genetic diversity. Note that, in practice, the number of ideal-genotype individuals is often insufficient to satisfy the selection requirements of the above formula. In such cases, individuals carrying relatively more target genes may be used as substitutes.

### Overall of existing schemes: cascading and non-cascading pyramiding schemes

We compared our novel scheme with that of two classical schemes proposed by Servin et al. [29]: cascading and non-cascading pyramiding schemes [Additional file 1, Fig. S1].

Cascading scheme involves performing only one cross per generation. This type has the maximum number of generations, which is * n-1*, because only one gene is accumulated in each generation. Conversely, the noncascading type performs as many crosses as possible for each generation. This type requires the minimum generations, which needs to be the smallest integer satisfying $T\ge\log_2(n)$, identical to that of our rotating scheme. According to Servin et al. (2004), the total number of pyramiding schemes based on their theory is:

3 $$\varphi \left( n \right) = \mathop \prod \limits_{k = 2}^n \left( {2k - 3} \right) = \left( {2n - 3} \right)\left( {2n - 5} \right) \cdots 1$$

where, $\:\mathrm{n}$ is the total number of target genes in the different breeds or lines.

### Simulation of genotype and phenotype data

Here, we simulated diploid animal populations based on a typical purebred pig breeding scenario. In this scenario, the target gene favorable variants were absent from the base population and introduced through gene editing prior to the pyramiding scheme. Traits 1 to 3 with different heritabilities were used as background traits, originating from age adjusted to 100 kg (AGE), back-fat thickness adjusted to 100 kg (BF), and total number of piglets born (TNB) in pigs. Genotype and phenotype data were simulated using the R package AlphaSimR (1.7.4) [32].

Following previous studies [33–36], the means of phenotypes were set to 180 (days), 20 (mm), and 10 (piglets); the variances of phenotypes were 80, 2, and 3; and the heritabilities were 0.3, 0.4, and 0.1, respectively. The genetic correlation between trait 1 and 2 is -0.3, whereas the genetic correlations between trait 3 and the other traits were zero.

The parameters were set as follows: The founder population was assumed to be non-inbred and diploid. The genome consisted of 18 pairs of autosomal chromosomes with 100 QTLs and 100 SNPs per chromosome. The genetic length of each chromosome was set to 100 cM, yielding a total genome size of 1800 cM. The founder population was generated using AlphaSimR’s coalescent simulation, which produces LD patterns consistent with mutation-drift equilibrium. LD between successive SNP markers was highly variable (median r² = 0.074), with most pairs showing weak LD but a subset exhibiting strong or perfect LD due to the coalescent demographic history. Similarly, LD between causal QTLs and the nearest SNP marker had a median r² of 0.098.

Recombination was modeled using the gamma distribution with default parameters [v = 2.6 (close to Kosambi function), *p* = 0], where v represents the crossover interference parameter and p denotes the proportion of crossovers from a non-interfering pathway.

### Simulation of estimated breeding value

To simplify the calculation, the estimated breeding values (EBVs) in this study were simulated based on the true breeding value (TBV) and the available empirical accuracy of EBV prediction, following the approach described by Eikje et al. [37]. The equation for simulating the EBV is:

4 $$\:{\mathrm{EBV}}_{\mathrm{sim}}=\mathrm{u}+{\mathrm{r}}^{2}\left(\mathrm{TBV}-\mathrm{u}\right)+\mathrm{r}\times\mathrm{s}\times\sqrt{1-{\mathrm{r}}^{2}}\times\mathrm{e}$$

where, $\:{\mathrm{EBV}}_{\mathrm{sim}}$ is the vector of the simulated $\:\mathrm{EBV}$ of all individuals, $\:\mathrm{u}$ is the mean of the $\:\mathrm{TBV}$ of all individuals, $\:\mathrm{r}$ is the accuracy (Pearson’s correlation coefficient between $\:\mathrm{TBV}$ and $\:\mathrm{EBV}$), $\:\mathrm{e}$ is a randomly generated vector following a normal distribution N (0,1), and $\:\mathrm{s}$ is the standard deviation of TBV (i.e. additive genetic standard deviation).

### Characterization of population diversity and inbreeding

For each generation, we calculated the inbreeding coefficient of the breeding population and the average minor allele frequency (MAF) of all loci to characterize the allelic diversity at each loci in the population. The inbreeding coefficient and genetic relationship was calculated from the pedigree with Wright’s formula [38] in the R package nadiv (2.17.3).

### Gene editing and selection scheme

Four scenarios were simulated in this study: three target gene preference scenarios (cascading, non-cascading, and mRMC) and one baseline scenario.

To simulate the breeding process of purebred pigs, an aggregate selection index ($\:\mathrm{I}$) was calculated based on the following equation:

5 $$\:I={w}_{1}{ebv}_{1}/{\delta\:}_{1}+{w}_{2}{ebv}_{2}/{\delta\:}_{2}+{w}_{3}{ebv}_{3}/{\delta\:}_{3}$$

where $\:{\mathrm{w}}_{\mathrm{1}}$, $\:{\mathrm{w}}_{\mathrm{2}}$ and $\:{\mathrm{w}}_{\mathrm{3}}$ are the selection weights for three traits and are set to be -0.4, -0.3, and 0.3, respectively, and ${{\rm{\delta }}_{\rm{1}}}$, ${{\rm{\delta }}_{\rm{2}}}$, and ${{\rm{\delta }}_{\rm{3}}}$ are the standard deviations of $\:\mathrm{ebv}$ for traits 1, 2, and 3, respectively. This implies negative selection was performed for traits 1 and 2, and positive selection for trait 3.

For the target gene preference scenarios, we randomly selected five target genes across the entire genome to ensure that they were located on different chromosomes [Additional file 3, Table S1]. To exclude any influence of target genes on background traits, the positions of target genes were set to be non-overlapping with quantitative-trait-locus (QTL) regions of background traits 1 to 3, with a minimum distance of 1 cM.

Here, we simulated diploid animal populations based on a typical purebred pig breeding scenario, where target gene favorable variants were absent in the base population and introduced through gene editing prior to the pyramiding scheme.

#### Base population and pre-editing selection

We first simulated five generations of genomic selection in a base population of 2000 individuals (1000 males and 1000 females) using the GBLUP (Genomic Best Linear Unbiased Prediction) model [39]. In each generation, individuals with the highest aggregate selection index calculated based on GEBVs were selected for breeding. GEBVs for each subsequent generation were predicted from the preceding generation, yielding mean accuracies of 0.72, 0.77, and 0.41 for the three background traits, respectively. These values were subsequently adopted as the GEBV accuracies in the following simulations.

#### Establishment of edited founder lines

From the fifth generation of the base population, 5 males and 5 females with the highest aggregate selection index were selected and subjected to gene editing to establish the initial paternal lines. The remaining individuals in the population did not carry any target genes. Target genes labelled 1–5 correspond to paternal lines with the same label number, with individuals in line $\:\mathrm{i}$ edited to be homozygous for the favorable allele of target gene $\:\mathrm{i}$. To simplify the selection process, a single perfect marker was designed for each simulated target gene.

Generation 0 (G0) of the following study comprised 5 gene-edited males and 5 gene-edited females together with individuals carrying no target genes. Including G0, every generation of the breeding process consisted of 50 sires, 500 dams, and 5000 offspring, with the 50 sires from 5 paternal lines and 10 sires per line. Each dam was mated to only one sire, and each sire was mated to 10 different dams. Each line generated 1000 offspring from 10 sires and 100 dams. The sex ratio of the progeny was 1:1, and the litter size was set to 10 in each mating pair.

In the baseline scenario, the breeding individuals were ranked based solely on the aggregate selection index. In the target gene preference scenarios, breeding individuals were firstly ranked based on the genotype of the target gene, retaining the following order: (1) number of target gene types, (2) total number of target genes carried, and secondly on aggregate selection index. Sibling mating was prohibited in all schemes. The selection process was conducted using in-house R scripts, with 100 repetitions.

#### Linkage disequilibrium between target genes and background QTLs

Although target genes were placed outside the predefined QTL regions for background traits, some linkage disequilibrium (LD) may still exist due to physical proximity on the same chromosome. We calculated the squared correlation (r²) between each target gene and all background QTLs on the same chromosome across the simulated generations. The majority of r² values were very low (median < 0.01), indicating that target genes were largely independent of the genetic architecture underlying background traits. However, a small number of QTLs showed moderate to high LD with target genes (maximum r² = 0.96 for target gene 1, 0.33 for target gene 4, and 0.20 for target gene 5). These associations are stochastic consequences of genome-wide sampling and do not reflect functional relationships, but they introduce minor noise into the selection for background traits. Given that genomic selection uses genome-wide markers rather than individual QTLs, the overall impact of these localized LD patterns on GEBV accuracy and genetic gain is expected to be small.

## Results

### Efficiency of pyramiding and fixation

A total of ten generations (abbreviated as G0–G9) were simulated in this study, encompassing the processes of pyramiding and fixation. The pyramiding step was defined as the period from the initial state to the first occurrence of an individual carrying all the target genes, and the completion of the fixed step was defined as all individuals in the population carrying all the favorable alleles of target genes.

**Fig. 3:**
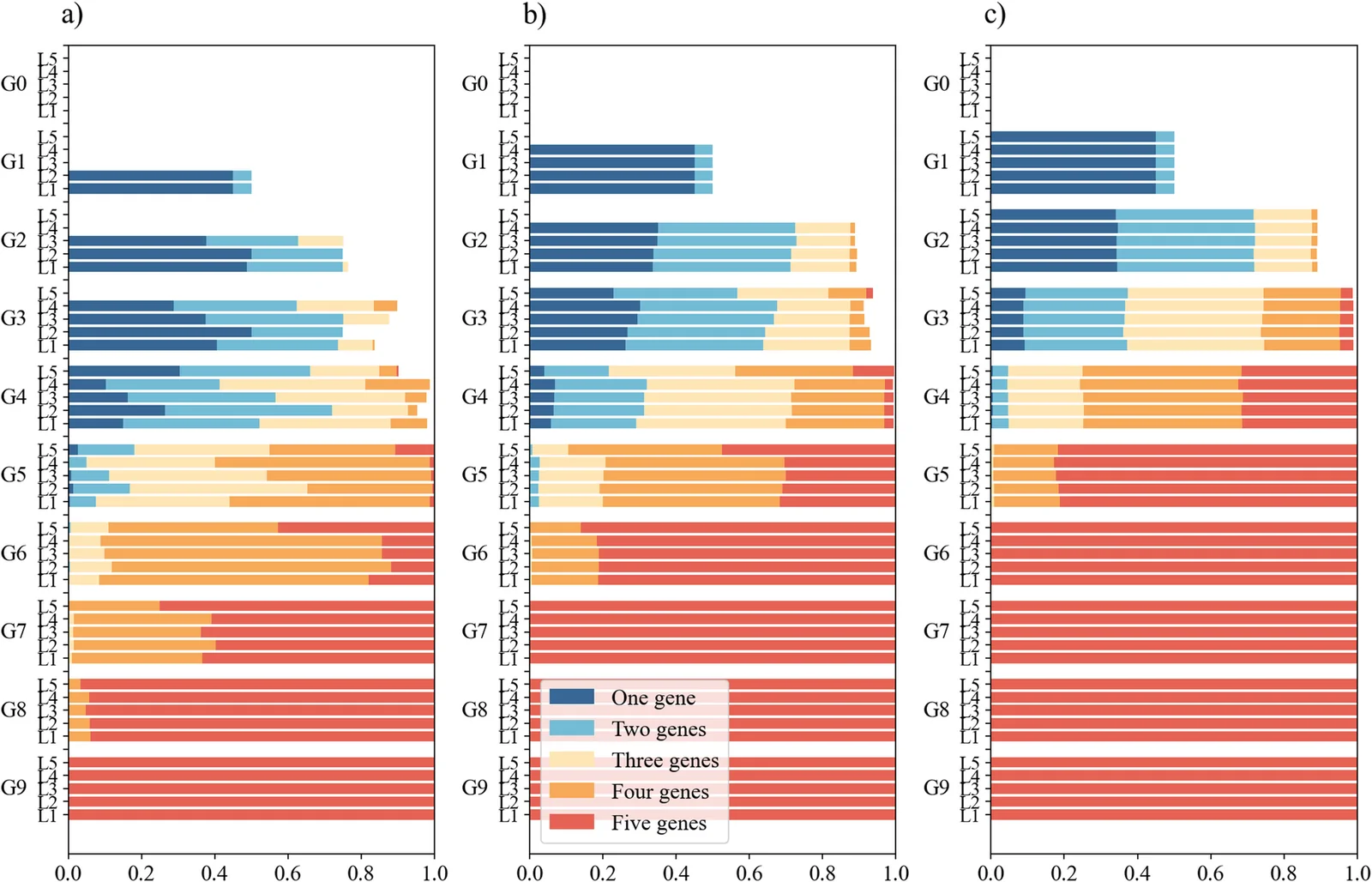
Proportion of individuals of each genotype in each generation of each linage. (a) cascading, (b) non-cascading, and (c) rotating schemes. The horizontal coordinate represents the proportion of individuals with specific genotype. Different colors indicate the number of target genes carried. The number of genes in the legend refers to the types of target genes an individual carries, rather than the total number. L1–L5: Labels of the five paternal lines. G0–G9: Serial number of generations from the start to the end

During the pyramiding step, the three schemes showed varying efficiencies for the proportion of individuals of each genotype in each generation [Fig. 3, Additional file 1, Fig. S4 and Additional file 3, Table S2]. For the mRMC and non-cascading schemes, five-target-gene-carriers were generated in G3, indicating completion of pyramiding. The total numbers of generations required in the cascading, non-cascading, and rotating schemes were four, three, and three, respectively. In each generation, mRMC consistently achieved the highest number of target genes in the population [Additional file 1, Fig. S2]. Under the mRMC scheme, 3.60% of the individuals were five-target-gene-carriers in G3, while the non-cascading scheme generated 0.38%. Although mRMC did not exhibit a time-cost advantage in the pyramiding step compared to that of the non-cascading scheme, it demonstrated a higher efficiency in gene spreading process (Fig. 3).

The total number of generations required in the fixation step was also counted. The mRMC was superior to those of the other schemes [Table 1 and Additional file 1, Fig. S2]. For mRMC, all individuals became five-target-gene-carriers in G6, implying that the fixation step was completed. The cascading and non-cascading schemes required five and four generations, respectively, whereas mRMC only used three generations of intercrossing to generate an ideal population, making it the most time-saving strategy.

**Table 1:** Number of generations required for obtaining the ideal population under different schemes

Scheme	Pyramiding Step	Fixation Step	Total
Cascading pyramiding	4	5	9
Noncascading pyramiding	3	4	7
Rotating pyramiding (mRMC)	3	3	6

This table shows the number of generations of pyramiding and fixation, and the total number of generations of these two steps for obtaining the ideal population under different schemes in our simulation study. The ideal population indicates all individuals in the population carrying all target genes. The pyramiding step indicates the period from the initial state to the first occurrence of an individual carrying all the target genes. The completion of the fixed step was defined as all individuals in the population carrying all the target genes.

### Total time consumption for pyramiding

During gene pyramiding, breeders aim to maximize the efficiency of each mating by selecting pairs whose combined target genes exhibit the minimum intersection and maximum union. In information theory terms, this is equivalent to choosing two messages with the lowest mutual information and the highest joint entropy, thereby maximizing the efficiency of information transmission per generation. Under mRMC, the total number of generations required for pyramiding is the minimum integer that satisfies $T \ge \log_2(n)$, where $\:\mathrm{n}$ is the total number of target genes, which is equivalent to the non-cascading pyramiding scheme. These two schemes exhibit identical pyramiding efficiencies, and both achieve the highest efficiency from the perspective of information theory. While, under the cascading pyramiding scheme, cross or backcross is allowed to be performed only once per generation, thus the total number of generations for pyramiding is $\:\mathrm{T}\mathrm{=}\mathrm{n}\mathrm{-1}$. Obviously, when $\:\mathrm{n}$ ≥ 4, $\log_2(n)$ is always less than $\:\mathrm{n}\mathrm{-1}$.

### Contribution of gene-edited individuals to ideal individuals

It can be measured by the number of individuals required in the pyramiding steps, i.e., the total number of individuals needed throughout the entire process to produce one ideal individual. Assuming that each target gene was located on a different chromosome and was consistent with Mendelian laws of inheritance, we calculated the minimum population size at each node based on the formula used by Servin et al. [29] [Table 2]. Here, we focused on the number of gene-edited individuals required under the different schemes. When both males and females participated in gene editing, the numbers of gene-edited individuals needed for cascading, non-cascading, and mRMC were 540, 25, and 21, respectively. When only males are involved in gene editing, the numbers were 1605, 15, and 4, respectively, indicating that the efficiency of mRMC in spreading target genes was higher than that of the others.

**Table 2:** Minimum population sizes necessary to obtain at least one ideal individual under different schemes

	Schemes	Generation					Number of gene-edited individuals	Total population size
		G0	G1	G2	G3	G4		
Scenario 1	Cascading	28.9	205.2	704.5	70.5		539.7	1000.0
	Noncascading	13.5	91.4	18.3			25.0	123.2
	Rotating (mRMC)	7.8	105.9	21.2			21.2	134.9
Scenario 2	Cascading	58.2	840.8	5973.1	9519.3	1903.9	1604.5	20990.4
	Noncascading	27.5	185.8	563.5	112.7		14.5	1018.8
	Rotating (mRMC)	7.8	105.9	321.1	64.2		3.7	572.2
This table shows the minimum population sizes in each generation under three schemes and two scenarios. The population sizes were calculated based on the equation${\rm{N = }}{{{\rm{ln(1 - \gamma )}}} \over {{\rm{ln(1 - p)}}}}$in which N is the minimum population size needed to get the intermediate genotype at this node with a probability${\rm{\gamma }}$, p is the probability of producing a specific genotype at this node. The overall probability of success was set to 0.95. Scenario 1 indicates that both females and males were gene edited. Scenario 2 indicates that only males were edited, which is a common strategy in breeding industry.								

### Population inbreeding and genetic diversity

**Fig. 4:**
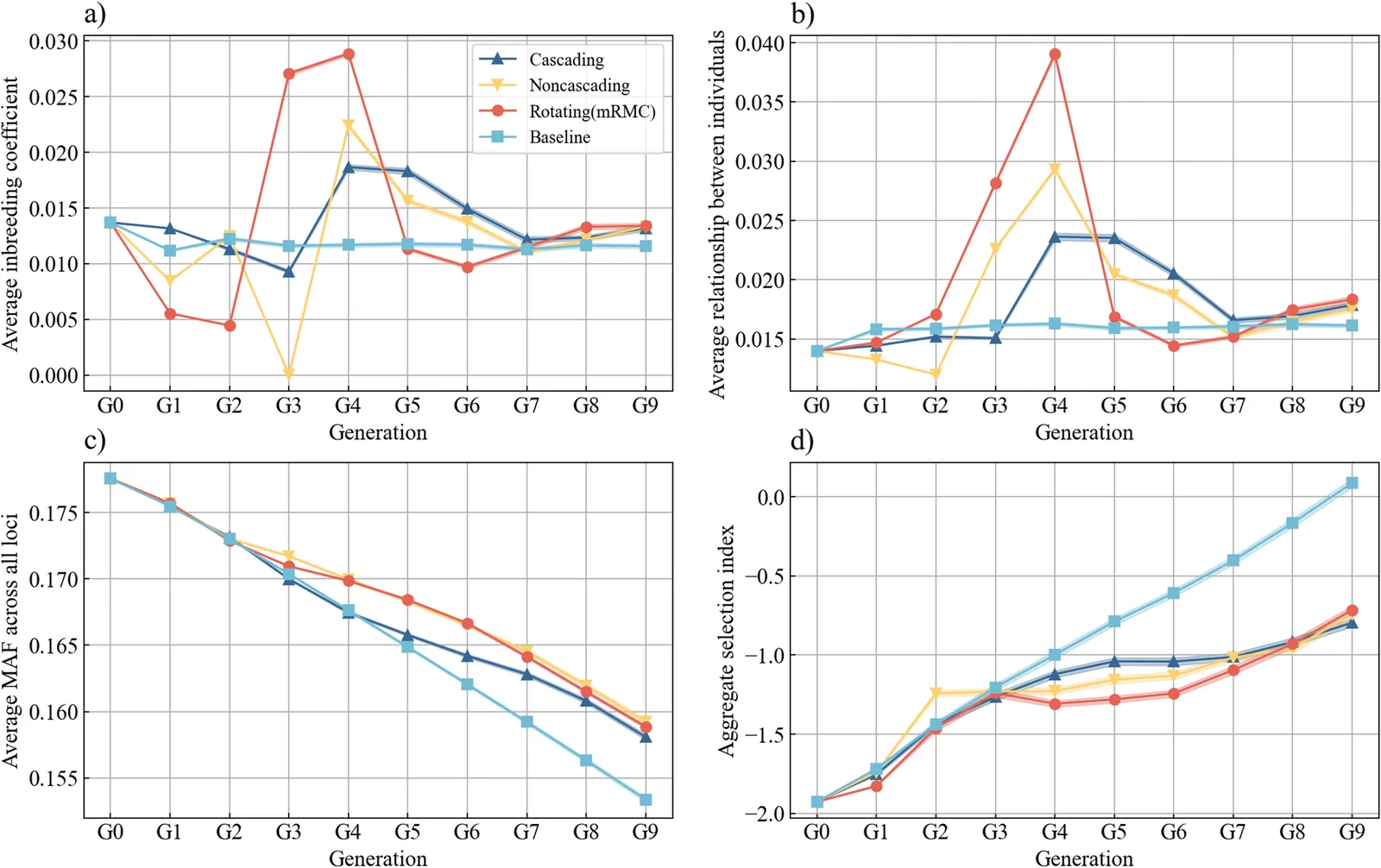
Tendency of population parameters under different schemes. The horizontal coordinate represents the value of (**a**) the average population inbreeding coefficient, (**b**) relationship between individuals, (**c**) average MAF and (**d**) aggregate selection index of the population. The vertical coordinate indicates the generation number. The colors dark blue, yellow, red, and sky blue correspond to the cascading, non-cascading, rotating, and baseline schemes, respectively

For inbreeding levels, mRMC presented a lower trend than that of the other schemes during the pyramiding generations, and it was even lower than that of the baseline scheme throughout all generations in the pyramiding step (Fig. 4a). In the subsequent fixation step, inbreeding levels under mRMC tended to be more stable compared to those in other schemes, whereas the non-cascading scheme exhibited a sudden increase in G4, and the cascading scheme maintained a high level during the entire pyramiding process. Eventually, the inbreeding levels under all the schemes stabilized at about 0.013 (Fig. 4a).

The trend in average relationship was not fully aligned with inbreeding level (Fig. 4b), as average relationship reflects the inbreeding status of future generations rather than the current population. Especially for G3 of non-cascading scheme [Additional file 1, Fig. S1], inbreeding level sharply decreased due to the mating between different paternal lines, but in reality, the average genetic relationship between individuals increased.

The trends of average MAF across all loci of all individuals were similar across all schemes (Fig. 4c), which could be attributed to a decrease in genotype heterozygosity due to selection. mRMC demonstrated greater preservation of genetic diversity throughout all generations, providing more genetic variance for the subsequent breeding program.

### Genetic gain

Compared with that of the baseline scheme, which selects individuals based purely on the aggregate selection index, we observed that all schemes incorporating target gene pyramiding led to a significantly lower genetic gain as the number of generations increased [Fig. 4d, Additional file 1, Fig. S3a]. In the final generation, the genetic gain reached 1.13, 1.19, 1.33, 2.01 for cascading, non-cascading, mRMC and baseline, respectively. That is, relative to cascading and non-cascading, mRMC reduced the loss of genetic gain by 17.70% and 11.76%, respectively, though genetic gain remained below that of the baseline.

## Discussion

We propose a novel strategy, mRMC, to adapt to the characteristics of animals based on a rotating design for multi-gene pyramiding. Previous pyramiding studies focusing on animals provided the conceptual foundation for our study. Piyasatian et al. [40] proposed assembling favourable alleles by creating a synthetic population of the breeds that carry favorable alleles and then conducting MAS within this synthetic population to increase the frequency of favorable alleles. Zhao et al. [30] subsequently identified population size as the critical determinant of multi-gene pyramiding feasibility and emphasised the need for optimal cross selection. Although these studies did not introduce methodological innovations specific to outbred animal populations, they provided a reference basis for this study.

Subsequently, we investigated the application of the mRMC using simulation data. Compared to those of the traditional schemes based on the biological characteristics of plants [29], mRMC exhibited the highest pyramiding efficiency, while providing good performances in terms of genetic diversity, genetic gain and inbreeding trend.

Particularly, using the mRMC method, the pyramiding scheme can be uniquely determined based on the number of target genes, which eliminates the need to select a breeding plan and provides a one-size-fits-all approach, offering the possibility of computer-guided breeding.

### The genetic background composition of ideal individuals

Under mRMC, the genetic background of ideal individuals is always uniformly composed and derived from $\:\mathrm{n}$ paternal lines, with each line constituting $\:\mathrm{1/}\mathrm{n}$. In the cascading scheme, the genetic background is sourced from $\:\mathrm{n}$ paternal lines; however, the $\:\mathrm{nth}$ line occupies 1/2. For non-cascading scheme, if $\:\mathrm{n}$ is even, the genetic background is uniformly composed (if $\:\mathrm{n}$ is divisible by 4) or the $\:\mathrm{(}\mathrm{n}\mathrm{-1)}\text{}\mathrm{th}$ and $\:\mathrm{n}\mathrm{th}$ line occupying 1/4, respectively (if $\:\mathrm{n}$ is not divisible by 4), if $\:\mathrm{n}$ is odd, the ancestral contribution consists of the $\:\mathrm{nth}$ line occupying 1/2 (Fig. 2 and Additional file 1, Fig. S1).

### Additional advantages of mRMC in fixation and population structure

For the fixation step, the population created under mRMC achieved a higher frequency of target genes, ultimately increasing fixation efficiency due to a more uniform distribution of target genes and genetic background composition. Contrastingly, existing schemes focus exclusively on pyramiding target genes and pay no regard to the dynamics of background traits and genetic structure of the population during the process.

Crossbred breeding between paternal lines results in a fluctuating inbreeding level during the pyramiding step, which tends to stabilize during the subsequent intercross fixation step. Notably, in this study, the population inbreeding level was indirectly controlled by maintaining the number of paternal lines across all four schemes, which is a common method used in commercial breeding. Thus, the superiority of mRMC is not very outstanding.

The average MAF under mRMC was even 4.76% higher than that of the baseline in the final generation (Fig. 4c), and the genetic variance of the three background traits was higher than that of the other two schemes in G3, i.e., the final generation of pyramiding [Additional file 1, Fig. S3b], indicating that mRMC behaved well in maintaining high genetic diversity and providing high genetic variance for subsequent generations.

### Limitations of the simplified simulation

#### Simplified genomic prediction

The simulation of background trait EBVs significantly reduced the computational complexity, and this simulation approach was similar to that of an earlier study based on gene editing in dual-purpose chicken [41]. It was suggested that an increased reference population in later generations would result in higher prediction accuracy. In this study, considering the reduced additive genetic variance through selection and the limited number of simulated generations, we set a fixed EBV prediction accuracy for each trait.

#### Linkage between target genes and background QTLs

Although target genes were placed outside predefined QTL regions, stochastic LD between target genes and background QTLs on the same chromosome introduced minor noise into selection for background traits. However, the median r² was below 0.01 for all target genes, suggesting this effect was small.

The pyramiding of target genes is an additional breeding objective based on the selection of background traits. The simulation results suggest that multiple breeding objectives hindered the genetic progress of background traits. The present study adopts a simplified scenario in which the modification of target genes is assumed to exert no influence on the expression of background trait. Empirical conditions are undoubtedly more complex: if target alleles confer favorable effects on background performance, gene pyramiding will additionally accelerate genetic gain; conversely, if there is a negative effect, the improvement of population production performance will be more hindered, attributed to the trade-off between the two breeding objectives.

#### Idealized founder population

The simulation adopted an idealized scenario in which the gene-edited initial generations of all paternal lines were already homozygous for the favorable alleles. This omits more realistic settings such as variable population sizes, imperfect editing efficiencies, incomplete genotype fixation, and segregating initial allele frequencies within each paternal line. As underlined by Xu et al. [31], this initial phase of establishing edited founder lines should be accounted for in a complete breeding scheme simulation. Incorporating these factors would likely extend the total duration of the pyramiding program and may increase the initial inbreeding bottleneck.

#### Trade-off between breeding objectives

Overall, the mRMC scheme demonstrated lower losses in genetic gain for the aggregate breeding goal compared with the other pyramiding strategies, attributable to its structured rotational design that maintains genetic diversity and selection intensity. This compromise reflects the inherent trade-off between gene fixation and genetic progress in multi-gene pyramiding programs. While the aggregate selection index provides a practical metric for routine breeding decisions, it does not fully capture the long-term genetic architecture improvements achieved through pyramiding.

### Positioning mRMC in the context of animal breeding practice

Although this study is a proof-of-concept, it is worth considering how mRMC might fit into existing breeding programs as biotechnology develops. Modern livestock breeding already uses structured paternal lines in nucleus herds, where separate lines are kept to make use of heterosis and manage genetic diversity. If gene-edited lines carrying different favorable alleles were developed—whether for disease resistance, production efficiency, or climate adaptation—mRMC provides a clear protocol for rotating these lines through a common nucleus population. The “minimum rotation, maximum complementarity” principle directly addresses a practical problem: breeders cannot keep increasing the number of separate edited lines forever without facing high costs and inbreeding. By systematically cycling lines and monitoring genetic diversity, mRMC offers a balance between (i) traditional introgression, which can cause severe bottlenecks and inbreeding depression, and (ii) random crossing of edited lines, which does not control genetic diversity. For local breeds carrying unique adaptive alleles, mRMC also provides an alternative to traditional backcrossing, keeping a larger part of the donor genome while gradually moving toward a multi-gene carrier population. However, we emphasize that the current simulation uses idealized conditions—homozygous edited founders, perfect markers, and fixed population sizes—that do not match actual on-farm conditions. In addition, the population size required for the scheme was not addressed and may further constrain its practical application. Because most parents are heterozygous for the target genes, the probability that a single offspring inherits all target genes decreases rapidly as the number of target genes increases. A systematic evaluation of the minimum population size and selection intensity required under realistic reproductive scenarios is therefore an important next step before mRMC can be implemented in practice. The approximately 30–40% reduction in genetic gain for background traits is a theoretical trade-off whose economic value depends on the specific importance of the pyramided genes. Therefore, mRMC should be seen as a conceptual contribution to the methods for future gene pyramiding, rather than a protocol ready for immediate use.

### When and how might mRMC be used in commercial breeding?

Whether mRMC is useful depends on the economic and biological importance of the target alleles. We consider three situations. First, resistance to severe diseases: when a major resistance allele becomes available (e.g., CD163 knockout for PRRSV resistance in pigs [11, 16] ), the losses from not using it may exceed the genetic gain sacrificed during pyramiding. A breeder could edit one of five paternal lines and use mRMC to rotate it through the nucleus population over six generations, reaching fixation while controlling inbreeding. Second, combining production alleles: if several confirmed QTL for feed efficiency or meat quality were found in different lines, mRMC could bring them together systematically, although the breeder must judge whether the extra gain is worth the delay. Third, traits driven by regulations or markets: when consumer demand or rules require adopting a welfare-related trait (e.g., polled genetics in cattle [17]), pyramiding may be the only way to introduce it without losing existing genetic progress.

In all cases, the practical steps would be setting up edited founder lines, performing the mRMC rotation strategy with concurrent genomic selection for background traits, tracking inbreeding and MAF, and switching to normal selection once fixation is done. The main consequence is a temporary drop in genetic gain during pyramiding and a one- to three-generation lag before multipliers can spread the improved genetics. This delay may be acceptable for high-value traits but too costly for minor production traits.

## Conclusions

We proposed a novel gene pyramiding strategy, mRMC, for outbred animals and demonstrated its effectiveness in a simulation study based on animals. Compared with other original schemes, mRMC outperformed in terms of the pyramiding efficiency, genetic gain, and genetic diversity. Gene pyramiding in outbred animals is a practical challenge that sits between gene discovery and applying results at the population level. Our mRMC scheme provides a proof-of-concept framework that balances pyramiding efficiency with maintaining genetic diversity, offering a structured alternative to existing strategies developed for plants. While immediate application awaits further advances in gene-editing technology, regulatory frameworks, and economic evaluation, we believe mRMC contributes a forward-looking perspective to the evolving toolkit for designed breeding in animals.

## Supplementary Information

Below is the link to the electronic supplementary material.

Supplementary Material 1

Supplementary Material 2

Supplementary Material 3

Supplementary Material 4

Supplementary Material 5

Supplementary Material 6

## Data Availability

The source data and scripts for simulation in this article are available in the repository: https://github.com/CAU-TeamLiuJF/RotatingPyramiding.
