# Supplementary material for: mRMC: A rotating multi-gene pyramiding scheme based on the principle of minimum rotation and maximum complementarity in animals
Source: Genet Sel Evol. 2026 Nov 11;58:86. doi: 10.1186/s12711-026-01112-2 (PMC13757030; doi:10.1186/s12711-026-01112-2)
Supplement: Supplementary file 1 [file 12711_2026_1112_MOESM5_ESM.docx]

**Supplementary material**

Figure S1 Examples of cascading (left) and noncascading (right) pyramiding scheme cumulating five target genes

Each circle represents one family, with the arrow indicating the mating strategy. The numbers within the circles represent the labels of the target genes. The proportion of bloodline composition of individuals carrying the target gene is represented by the area ratio of different colors in the circle.

Figure S2 Frequencies of favorable alleles of target genes among generations

The horizontal coordinate represents the frequencies of target gene, which is calculated as the sum of the five target genes to be divided by the total number of the five gene locus in the population, and the vertical coordinate indicates the generation. When the frequency reaches 1, it means that all individuals become ideal individuals. Dark-blue upright triangles, yellow inverted triangles, red circles and sky-blue squares denote Cascading, Noncascading, Rotating (mRMC) and baseline, respectively.

Figure S3 The tendency of genetic value and additive variance under different schemes of three background traits

The horizontal coordinate represents a) the mean of genetic value and b) the additive variance, which are derived from trait 1 to 3 from left to right, and the vertical coordinate indicates the generation. Dark-blue upright triangles, yellow inverted triangles, red circles and sky-blue squares denote Cascading, Noncascading, Rotating (mRMC) and baseline, respectively. The shaded area in the figure represents the standard deviation of the data.

Figure S4 The proportion of individuals of every genotype in each generation

The horizontal coordinate represents the proportion of specific genotype individuals. Different colors represent the number of target genes carried as shown in the caption. Number of genes in the legend represent how many type of favorable variants of target genes the individual carrying, rather than the total number. L1-L5: labels of the five families. S1: Cascading scheme, S2: Noncascading scheme, S3: Rotating scheme (mRMC). G0-G9: serial number of generations from the beginning to the end.
