# Supplementary material for: mRMC: A rotating multi-gene pyramiding scheme based on the principle of minimum rotation and maximum complementarity in animals
Source: Genet Sel Evol. 2026 Nov 11;58:86. doi: 10.1186/s12711-026-01112-2 (PMC13757030; doi:10.1186/s12711-026-01112-2)
Supplement: Supplementary file 2 [file 12711_2026_1112_MOESM6_ESM.docx]

**Supplementary material**

Table S1 Location of the five target genes on the genome

| Id | Chromosome | Site |
| --- | --- | --- |
| Gene 1 | 6 | 8448 |
| Gene 2 | 15 | 4791 |
| Gene 3 | 5 | 9570 |
| Gene 4 | 18 | 5845 |
| Gene 5 | 3 | 9497 |

This table shows the location of the five target genes selected in this study. The five genes were selected randomly on the whole genome excluding the QTLs of background traits. Gene 1-5: The label number of the target genes.

Table S2 Frequencies of **favorable alleles of** target gene among generations

|  | G0 | G1 | G2 | G3 | G4 | G5 | G6 | G7 | G8 | G9 |
| --- | --- | --- | --- | --- | --- | --- | --- | --- | --- | --- |
| S1 | 0 | 2.20% | 6.60% | 11.44% | 26.15% | 47.65% | 67.17% | 80.31% | 99.87% | 1 |
| S2 | 0 | 4.40% | 13.00% | 19.38% | 37.65% | 60.47% | 82.45% | 97.75% | 1 | 1 |
| S3 | 0 | 5.50% | 16.27% | 33.33% | 55.46% | 79.88% | 96.97% | 1 | 1 | 1 |
| S4 | 0 | 0 | 0 | 0 | 0 | 0 | 0 | 0 | 0 | 0 |

This is a supplement of Figure S2. The frequency is calculated as the sum of the favorable alleles of all the five target genes to be divided by the total number of the five gene locus in the population, and the vertical coordinate indicates the generation. S1: Cascading scheme, S2: Noncascading scheme, S3: Rotating scheme (mRMC) , S4: Baseline. G0-G9: serial number of generations from the beginning to the end.
